# Control of Leg Movements Driven by EMG Activity of Shoulder Muscles

**DOI:** 10.3389/fnhum.2014.00838

**Published:** 2014-10-20

**Authors:** Valentina La Scaleia, Francesca Sylos-Labini, Thomas Hoellinger, Letian Wang, Guy Cheron, Francesco Lacquaniti, Yuri P. Ivanenko

**Affiliations:** ^1^Laboratory of Neuromotor Physiology, Santa Lucia Foundation, Rome, Italy; ^2^Centre of Space Bio-Medicine, University of Rome Tor Vergata, Rome, Italy; ^3^Laboratory of Neurophysiology and Movement Biomechanics, Université Libre de Bruxelles, Brussels, Belgium; ^4^Department of Biomechanical Engineering, University of Twente, Enschede, Netherlands; ^5^Department of Systems Medicine, University of Rome Tor Vergata, Rome, Italy

**Keywords:** arm–leg co-ordination, quadrupedal locomotion, EMG patterns, gait kinematics, neuroprosthetic technology

## Abstract

During human walking, there exists a functional neural coupling between arms and legs, and between cervical and lumbosacral pattern generators. Here, we present a novel approach for associating the electromyographic (EMG) activity from upper limb muscles with leg kinematics. Our methodology takes advantage of the high involvement of shoulder muscles in most locomotor-related movements and of the natural co-ordination between arms and legs. Nine healthy subjects were asked to walk at different constant and variable speeds (3–5 km/h), while EMG activity of shoulder (deltoid) muscles and the kinematics of walking were recorded. To ensure a high level of EMG activity in deltoid, the subjects performed slightly larger arm swinging than they usually do. The temporal structure of the burst-like EMG activity was used to predict the spatiotemporal kinematic pattern of the forthcoming step. A comparison of actual and predicted stride leg kinematics showed a high degree of correspondence (*r* > 0.9). This algorithm has been also implemented in pilot experiments for controlling avatar walking in a virtual reality setup and an exoskeleton during over-ground stepping. The proposed approach may have important implications for the design of human–machine interfaces and neuroprosthetic technologies such as those of assistive lower limb exoskeletons.

## Introduction

Neuroprosthetic devices based on brain–machine interface (BMI) technology have the potential to restore mobility to both upper and lower extremities and to enable walking (Millán et al., 2010; Lebedev et al., 2011; Cheron et al., 2012; Wolpaw, 2013). Exoskeleton robotic devices are extensively developed in recent years to provide new possibilities for severely paralyzed patients to walk (Sale et al., 2012; Malcolm et al., 2013; Wang et al., 2013; Sylos-Labini et al., 2014b). The development of practical applications for gait assistance may involve a wide range of potential control implementations, from using trunk movements (Wang et al., 2013) or myoelectric signals (del-Ama et al., 2012; Alcaide-Aguirre et al., 2013; Gordon et al., 2013) to implementing BMI-based technology (Fitzsimmons et al., 2009; Gwin et al., 2011). To develop effective neuroprosthetic devices for human beings, BMI research has to address a number of issues related to improving the quality of neuronal recordings, and extending the BMI approach to a broad range of motor and sensory functions (Lebedev et al., 2011). In human beings, non-invasive electroencephalogram-based brain–machine interfacing for providing a reliable control of walking in the exoskeleton is still very limited (Gwin et al., 2011; Cheron et al., 2012; Wagner et al., 2012), and alternative or supplementary control strategies should be considered in parallel. The current study investigates a novel approach for associating the upper limb electromyographic (EMG) activity with leg kinematics. This approach holds promise for applications in the field of assistive lower limb exoskeletons.

The co-ordination of limb and body segments in locomotion arises from stereotyped coupling of cervical and lumbosacral spinal segment outputs (Ballesteros et al., 1965; Murray et al., 1967; Hogue, 1969; Wannier et al., 2001; Zehr and Duysens, 2004; Ivanenko et al., 2006; De Sèze et al., 2008; Barthelemy and Nielsen, 2010; Meyns et al., 2013; Sylos-Labini et al., 2014a). The co-ordination between arms and legs during human locomotion shares many features with that in quadrupeds (Falgairolle et al., 2006; Patrick et al., 2009; Juvin et al., 2012; MacLellan et al., 2012). Due to the natural arm–leg co-ordination in human walking, the leg movement control can be derived from or reflected in the EMGs of arm muscles. In particular, a group of the upper arm muscles was shown to be active during many locomotion tasks in human beings (Ivanenko et al., 2006; Kuhtz-Buschbeck and Jing, 2012). Taking into account, the high involvement of the deltoid muscle in most locomotor-related movements in human beings, we took advantage of the natural arm–leg co-ordination for investigating the control of stepping using the upper limb EMG activity. Here, we tested the hypothesis that the temporal structure of EMG activity of shoulder muscles can be used to predict the spatiotemporal kinematic pattern of the forthcoming step during actual gait at 3–5 km/h (and we investigated the accuracy of this prediction) and to control avatar walking in a virtual reality setup or an exoskeleton during over-ground stepping.

## Materials and Methods

### Participants and protocols

Two main protocols were implemented. In the first series of experiments performed in Brussels (protocol 1), we investigated the possibility of extracting lower limb kinematic data from the gait-related rhythmic EMG activity of arm muscles. To this end, nine healthy volunteers were enrolled [age range 23–33 years, four males and five females, leg length 0.85 ± 0.04 m (mean ± SD), height 1.75 ± 0.10 m, weight 72 ± 7 kg]. In the second series of experiments performed in Rome (protocol 2), we implemented the on-line control of avatar walking and also over-ground walking in the exoskeleton. For this protocol, eight healthy volunteers were enrolled (age range 25–53 years, five males and three females, leg length 0.83 ± 0.04 m, height 1.77 ± 0.09 m, weight 69 ± 8 kg). None of the subjects had any known neurological or motor disorder. These protocols were approved by the Ethics Committees of the Université Libre de Bruxelles (Belgium) and Fondazione Santa Lucia (Rome, Italy), respectively, and all subjects gave their informed written consent prior to participation.

### Experimental setup and data analysis

#### Protocol 1

In the first protocol, the subjects were asked to walk on a treadmill (Cosmed Treadmill T150) at different constant speeds (3, 4, and 5 km/h) and at a variable speed (ramp-and-hold velocity profile, increasing from 3 to 5 km/h at 0.1 km/h/s), four trials total (3 constant speeds + 1 trial at a variable speed). On average, 10–15 strides were recorded in each trial during walking at a constant speed (that corresponds to a walking distance of ~15–20 m) and about 50 strides during walking at a variable speed (distance ~70 m). During normal walking, the amplitude of arm swinging depends on the walking speed (Webb et al., 1994) and, at slow speeds, the activity of arm muscles decreases significantly (Ivanenko et al., 2006; Kuhtz-Buschbeck and Jing, 2012). Therefore, to obtain a higher level of arm EMG activity, the subjects were asked to perform slightly larger arm swinging than they usually do. In the preliminary training sessions (2–3 min of walking), we verified that these movements evoked consistent EMG activation of shoulder muscles at all walking speeds (3–5 km/h). We recorded kinematic data bilaterally at 100 Hz by means of the Vicon system (Vicon, Oxford, UK) with 10 Bonita cameras spaced around the treadmill. Infrared reflective markers (diameter 15 mm) were attached on each side of the subjects to the skin (using Vicon BioMind asymmetric model), in particular, overlying the following landmarks used to calculate leg kinematics: greater trochanter (GT), lateral femur epicondyle (LE), lateral malleolus (LM), and fifth metatarsophalangeal joint (VM). EMG activity of the anterior deltoid (DELTa) and posterior deltoid (DELTp) muscles was recorded bilaterally by means of surface electrodes. The EMG data were recorded with the wireless BTS Freeemg system (BTS Bioengineering, Milano, Italy) and digitized at 1000 Hz. Sampling of kinematic and EMG data were synchronized.

#### Data processing

**Figure 1:**
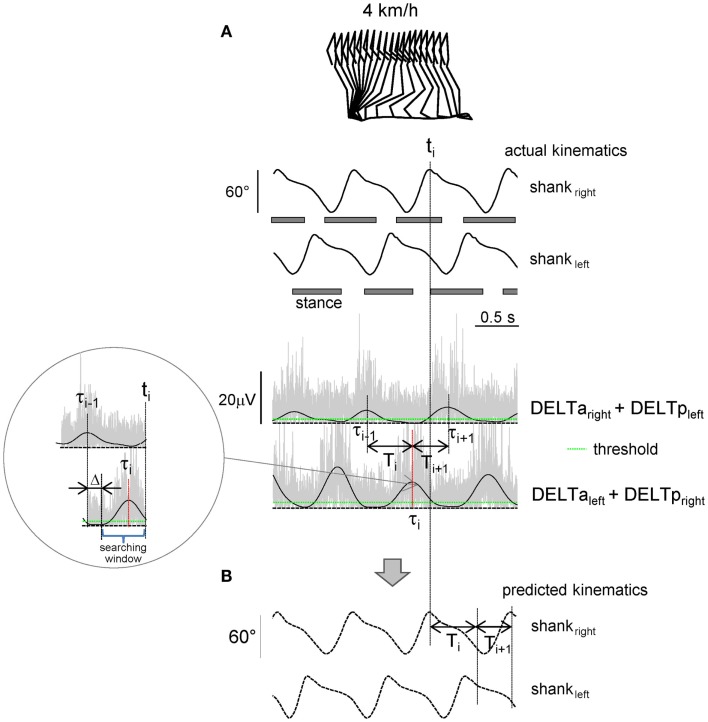
**Shoulder muscle EMG-based prediction of stepping kinematics**. **(A)** Schematic algorithm. Upper traces – actual left and right shank elevation angles during three consecutive strides. Lower traces – rectified (gray) and low-pass filtered (2 Hz, black) EMGs of shoulder muscles. The algorithm consisted in searching the EMG peak (τ*_i_*) at the end of each step (*t_i_*) during the appropriate time window [(τ*_i_*_−1_ + Δ, *t_i_*), see insert] that exceeded the pre-defined individually adjusted threshold (green line). Each step duration (*T_i_, T_i_*_+1_, etc.,) was predicted from the timing of the shoulder EMG peaks (*T_i_* = τ*_i_ −* τ*_i_*_−1_). **(B)** Predicted kinematic patterns.

Our approach uses the timing of the burst-like EMG activity of shoulder muscles (by applying the peak detection algorithm) to predict the spatiotemporal kinematic pattern of the forthcoming step. Prior to application of the peak detection algorithm, the EMG data were pre-processed: high-pass filtered at 30 Hz, rectified, and finally low-pass filtered (all filters, zero-lag fourth order Butterworth). Low-pass filtering was performed at different frequencies (1 ÷ 5 Hz) to achieve the best correlation between actual and predicted leg kinematics. Despite some inter-individual variability, periods of EMG activity of DELTa and DELTp during normal walking tend to be alternating and correspond to those of the contralateral upper limb (Ivanenko et al., 2006; Kuhtz-Buschbeck and Jing, 2012), as well as multi-muscle synergy-based control interface may be more efficient than a single-muscle control (Lunardini et al., 2014). Therefore, bilateral EMGs of synergistic muscles (Ivanenko et al., 2006; Kuhtz-Buschbeck and Jing, 2012) were summed (Figure 1A):

(1) $$\begin{array}{ll} {\mathrm{EMG}}_{1} & ={\mathrm{DELTa}}_{\mathrm{right}}+{\mathrm{DELTp}}_{\mathrm{left}} \end{array}$$

(2) $$\begin{array}{ll} {\mathrm{EMG}}_{2} & ={\mathrm{DELTa}}_{\mathrm{left}}+{\mathrm{DELTp}}_{\mathrm{right}} \end{array}$$

the peaks of EMG_1_ and EMG_2_ occur around the beginning of the swing phase of the right and left legs, respectively (Figure 1A). Nevertheless, we also compared the performance of our algorithm using all four muscles (Eqs 1 and 2) and only pairs of contralateral muscles (DELTa_left_ and DELTa_right_; DELTp_left_ and DELTp_right_).

The elevation angles of the thigh (GT-LE), shank (LE-LM), and foot (LM-VM) segments (Lacquaniti et al., 2002; Cheron et al., 2012) were calculated as the angles between the segment projected on the sagittal plane and the vertical (positive in the forward direction, i.e., when the distal marker falls anterior to the proximal one). We divided the recorded kinematic and EMG data into gait cycles (maximum shank elevation angle as the beginning of the gait cycle), then interpolated each stride to 200 time points, and finally averaged across gait cycles (for each trial). These ensemble-averaged (thigh, shank, and foot) elevation angles were used as a reference (template) for predicting leg kinematics from shoulder muscle EMGs. The amplitude of arm oscillations was assessed as the peak-to-peak amplitude of anterior–posterior wrist marker movements relative to the shoulder averaged across both (left and right) sides of the body and across all strides of the trial.

#### Prediction of leg kinematics based on shoulder muscle EMG activity

The algorithm used to predict the kinematics of the following step from the temporal structure of pre-processed EMG activity is schematically depicted in Figure 1. In essence, it predicts stride duration from the temporal distance between EMG peaks, and the kinematic pattern of a reference template (derived from averages of the collected data) is then temporally stretched to fit the predicted duration so as to generate the predicted segment elevation angles. The algorithm started from the second recorded step and consisted in detecting the timing of the EMG_1_ or EMG_2_ peak (exceeding the pre-defined threshold) in the appropriate time window and associating it with the duration and kinematics of the following step. In particular, at the end of each predicted step (*t_i_*), the program computed the timing of the latest peak of EMG_1_ or EMG_2_ depending on the associated time lag (τ*_i_*, Figure 1A) and determined the duration of the following step:

(3) $$\begin{array}{llll} T_{i} & =\tau_{i}-\tau_{i-1}\; & & \;\\ T_{i+1} & =\tau_{i+1}-\tau_{i}\; & & \;\\ & \ldots \end{array}$$

The peak detection threshold was defined individually for each trial and each EMG as

(4) $$\mathrm{threshold}=k\times \mathrm{mean}\left(\mathrm{EMG}\right)$$

where mean (EMG) is the mean (EMG_1_) or mean (EMG_2_) over the trial, and *k* is the coefficient found empirically (*k* = 0.8) to provide the best detection of peaks.

The searching window started with a fixed delay Δ = 200 ms from the last EMG peak (Figure 1A) in order not to capture extra peaks (from the previous step). The kinematic pattern of the forthcoming step was calculated by scaling (stretching) the kinematic reference template (see above) according to the predicted *T_i_*. The flexibility in the timing of the EMG peak due to accelerating/decelerating arm movements allowed gradual changes in the predicted cycle duration. On the other hand, in the case of the absence of the detected EMG peak at the time *t_i_*, the algorithm did not produce further steps and was terminated (in such case the trial was not considered to be successful for the prediction of leg kinematics).

#### Protocol 2

In the second protocol, we implemented the on-line control of leg kinematics based on rhythmic activity of shoulder muscles in the virtual reality setup (eight subjects) and during walking in the exoskeleton (one subject). EMG activity of DELTa and DELTp muscles on each side was registered at 500 Hz by means of surface electrodes with the wireless Delsys Trigno EMG system (Delsys Inc., Boston, MA, USA), bandwidth of 20–450 Hz, and overall gain of 1000. LabView software was used to collect and process the EMG data and to predict leg kinematics in real time. The predicted kinematic signals (thigh, shank, and foot elevation angles) were sent at 100 Hz using the UDP protocol to the virtual environment (using XVR software of VRMedia S.r.l.) to animate walking avatar (third person viewpoint). The algorithm was similar (Figure 1, the low-pass filter was performed at 2 Hz, based on the results of the first protocol, see Results) although, in addition to the on-line step-by-step control of walking kinematics, it also included gait initiation and gait termination steps. The threshold for EMG peak detection (Eq. 4) was adjusted individually for each subject, computing the mean EMG activity during alternating arm movements at a self-selected rhythm in the preliminary trial. The subjects (in standing position) were instructed to swing their arms at approximately the same amplitude at self-selected, slow, and fast frequency, as well as at a variable frequency. We instructed the subjects to vary the frequency approximately in the range between the slow and fast frequencies performed in the previous trials. The duration of each trial was 1 min and three trials were performed at each arm frequency.

In a pilot experiment, one subject was also trained to control an exoskeleton during over-ground stepping along an 8-m walkway. The detailed description of the exoskeleton (called MINDWALKER, https://www.mindwalker-project.eu) is provided elsewhere (Wang et al., 2013). Briefly, knee and hip exoskeleton joints (powered by series elastic actuators) followed pre-defined joint angles (based on the walking patterns of the same subject walking in the MINDWALKER exoskeleton without assistance) provided with variable joint impedances (Wang et al., 2013). The algorithm was similar to that used in the first protocol and detected the timing of EMG peaks. The output of the LabView software triggered and determined the initiation and the duration of the swing phase of each leg.

### Statistics

Descriptive statistics included means ± SD of the mean. The efficiency and accuracy of the predicting algorithm during walking at a constant speed (3, 4 and 5 km/h) were assessed using two parameters: the number of successful trials (if 10 consecutive strides were successfully predicted) and correlation between predicted and actual segment elevation angles. The efficiency of the method during walking at a variable speed (3–5 km/h) was assessed using the correlation between predicted and actual segment elevation angles, and the correlation between predicted and actual stride durations. In protocol 2, since the actual leg kinematics was not performed, the efficiency of the on-line algorithm using a virtual reality setup was assessed by the percentage of successful trials (if the algorithm produced alternating uninterrupted steps during the 1-min trial) during slow, self-selected, and fast arm movements. Statistics on Pearson’s correlation coefficients was performed on the normally distributed, *Z*-transformed values.

A repeated measure (RM) ANOVA was used to evaluate prediction (correlation coefficients between predicted and actual limb segment elevation angles), and *post hoc* Tukey’s HSD test was used to determine statistical significance. In one subject, the algorithm failed to predict limb kinematics (presumably due to low-EMG activity, see Results) and his data were not included. In another subject, it failed at a high speed (5 km/h) and the missing data for this condition for the ANOVA were replaced by the unweighted mean value estimated from all other subjects. Reported results are considered significant for *p* < 0.05.

## Results

### Shoulder muscle activity during walking

The shoulder muscles we monitored (bilateral anterior and posterior deltoid) showed rhythmical EMG signals during walking in all subjects (on average, the amplitude of the main rectified EMG peak was 7.0 ± 1.7 μV, all muscles and all speed being pooled together), although in a few cases their activity was small (<3 μV). Since the subjects were asked to perform approximately the same amplitude of reciprocal arm swinging [forward arm swing reverses to backward arm swing in the middle of the gait cycle (Kuhtz-Buschbeck and Jing, 2012)], EMG activity did not decrease with decreasing walking speed, as it normally occurs during walking (Ivanenko et al., 2006). On average, the anterior–posterior arm (wrist marker) oscillations were 50 ± 12 cm at 3 km/h, 45 ± 9 cm at 4 km/h, and 41 ± 10 cm at 5 km/h (for instance, during normal walking at 4–5 km/h, arm oscillations are ~35–40 cm, Murray et al., 1967; Webb et al., 1994; Ford et al., 2007), and the mean amplitude of deltoid muscle EMGs was 7.6 ± 1.7 μV at 3 km/h, 7.0 ± 1.7 μV at 4 km/h, and 6.1 ± 1.3 μV at 5 km/h.

**Figure 2:**
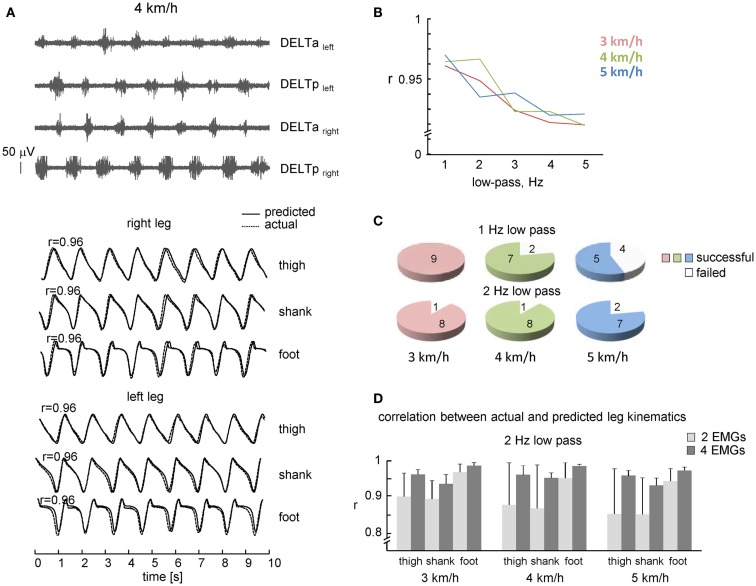
**Performance of leg kinematics prediction algorithm using shoulder muscle EMGs during walking at constant speeds**. **(A)** An example of muscle activity and kinematic patterns of one subject during walking at 4 km/h. Note, a fairly good correspondence between predicted (solid lines) and actual (dotted lines) thigh, shank, and foot elevation angles. **(B)** Correlation coefficients (averaged across all steps and subjects) between predicted and real shank segment elevation angles using different cut-off frequencies of low-pass filter. **(C)** Pie charts showing the percentage of subjects with a successful 10 consecutive strides prediction from shoulder EMGs activity (both 1 and 2 Hz low-pass filter for each speed). **(D)** Correlation (+SD) between actual and predicted kinematic patterns of individual subjects (2 Hz low-pass) using two and four shoulder muscle EMGs. Note, better predictions when using four EMGs.

Figure 2A illustrates an example of shoulder muscle EMG signals during walking at 4 km/h. Typically, the deltoid muscle demonstrated alternating activity during walking: alternation occurred both between left and right sides of the body and between anterior and posterior bellies of the deltoid (DELTa and DELTp). However, there could be an additional smaller second burst of activity over the gait cycle, as well as some inter-individual variability in the timing of the main EMG bursts [see also Ballesteros et al. (1965), Hogue (1969), Ivanenko et al. (2006), Kuhtz-Buschbeck and Jing (2012)]. Nevertheless, in most cases, there were prominent peaks of EMG_1_ and EMG_2_ around the beginning of the swing phase of the right and left legs, respectively (Figures 1A and 2A), which allowed us to associate this phasic alternating pattern of the upper limb EMG activity during arm–leg co-ordination with the spatiotemporal pattern of gait kinematics.

### Predicting leg kinematics from shoulder muscle EMGs during walking

We used the rectified and filtered EMG data to detect the timing of shoulder muscle EMG peaks in the concomitant and previous steps (see Materials and Methods) to predict the limb kinematics in the forthcoming step. On the whole, the best correlation between actual and predicted kinematics was observed at 1–2 Hz low-pass filtering (*r* > 0.95, Figure 2B), with slightly lower percentage of successful trials at 1 Hz low-pass at faster walking speeds (Figure 2C, presumably because the cut-off frequency was close to the step cycle frequency and the algorithm failed to detect EMG peak in time and was terminated), so that in the following we will present the results obtained at 2 Hz low-pass.

The percentage of subjects with a successful prediction of consecutive strides (2 Hz low-pass) from shoulder EMGs activity is shown in Figure 2C (lower plots). In one subject, EMG activity was rather low and the algorithm failed to predict gait kinematics. Some variability in EMG amplitude is to be expected due to individual differences in skin impedance and/or individual differences in the upper limb muscle EMG patterns during walking (Kuhtz-Buschbeck and Jing, 2012). Nevertheless, in most subjects (eight out of nine), it was successful at all speeds of walking.

In addition to the high rate of prediction (Figure 2C), the correlation between predicted and actual leg kinematics in successful trials was also high (Figure 2D). Figure 2A (lower curves) illustrates an example of actual (dotted lines) and predicted (solid lines) leg segment elevation angles during walking at 4 km/h. Despite some discrepancies (small phase shifts in the kinematic patterns of individual steps), there was a fairly good correspondence to actual lower limb kinematics, and a 1:1 ratio between frequencies of predicted and actual steps (absence of step “doubling” and absence of missing steps). The high-correlation coefficients between actual and predicted limb segment elevation angles (*r* > 0.9, Figure 2D) suggest stability of phasic alternating pattern of the upper limb EMG activity across consecutive strides.

We compared the performance of our algorithm using all four muscles (Eqs 1 and 2) and only two contralateral muscles (DELTa_left_ and DELTa_right_; DELTp_left_ and DELTp_right_). In the latter case, correlation coefficients of two pairs of muscles were averaged, since in ~30% of trials only one pair of contralateral muscles could predict leg kinematics while another pair failed to do it due to the relatively low/variable activity. The RM ANOVA (2 number of muscles × 3 speeds × 3 segments) resulted in a significant effect for muscle [*F*(1,7) = 9.686, *p* = 0.017], segment [*F*(2,14) = 34.18, *p* < 0.00001] and muscle × segment interaction [*F*(2,14) = 9.888, *p* = 0.002]. *Post hoc* tests revealed that correlations were higher when using four EMGs and they were also higher for the foot elevation angle relative to the shank and thigh elevation angles (Figure 2D). Thus, both the percentage of successful trials and the correlation coefficients were higher when using four shoulder muscles for prediction compared with only two muscles.

**Figure 3:**
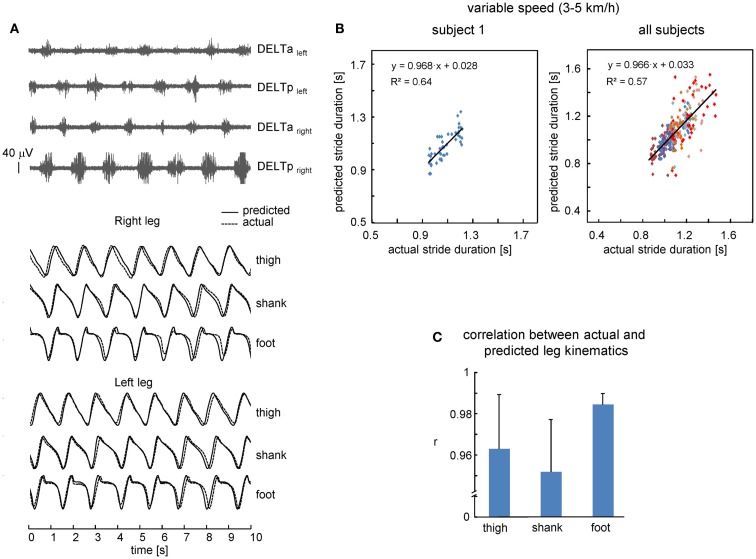
**Performance of leg kinematics prediction algorithm using shoulder muscle EMGs at variable walking speed (3–5 km/h)**. **(A)** An example of muscle activity and kinematic patterns of one subject during walking at a variable speed (ramp velocity profile, seven cycles in the middle of the trial are shown). **(B)** Relationship between predicted and actual stride duration for one representative subject (left) and all subjects (right). Each point corresponds to individual stride and data from each subject were displayed with different colors. Changes of predicted stride duration are fitted by a linear function. **(C)** Correlation (+SD, *n* = 8 subjects) between actual and predicted leg kinematics.

We also investigated the performance of the proposed leg kinematics prediction algorithm during walking at a variable speed (3–5 km/h). Figure 3B shows an example of cycle durations of all individual strides in one subject (left panel) and all successful (*n* = 8) subjects (right panel). The actual stride duration varied between 0.85 and 1.43 s, while the predicted stride duration also varied in a similar though slightly larger range (between 0.7 and 1.55 s, Figure 3B). The correlation between the reference template and individual cycles used for computing the template was high (on average 0.98 ± 0.01, the data for all segments were pooled together) consistent with a relatively low inter-stride variability of the segment elevation angles (Borghese et al., 1996; Bianchi et al., 1998). Therefore, inter-stride variability in the predicted stride durations and leg kinematics is likely related (at least in part) to inter-stride variability in the EMG patterns that has been documented for both leg and arm muscle activity during human walking (Kang and Dingwell, 2009; Kuhtz-Buschbeck and Jing, 2012; Zelik et al., 2014). On the whole, the relationship between predicted and actual stride duration was linear (Figure 3B) and correlation between predicted and actual leg kinematics was high (*r* > 0.95, Figure 3C). RM ANOVA resulted in a significant effect for segment [*F*(2,14) = 7.789, *p* = 0.0053] and *post hoc* tests revealed higher correlations for the foot elevation angle relative to the shank segment (*p* = 0.0045).

### Controlling virtual avatar and exoskeleton

**Figure 4:**
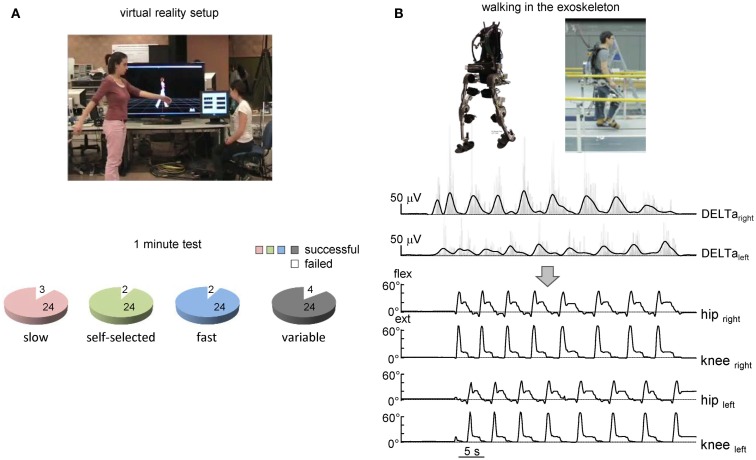
**On-line shoulder muscle EMG control of leg movements**. **(A)** Controlling of walking avatar in a virtual reality setup (third person viewpoint). To control the timing and duration of individual steps, the subject produced alternating arm swinging movements in standing position (upper panel). Lower panel – pie charts showing the percentage of trials with a successful 1-min test for producing stepping (if the algorithm predicted consecutive uninterrupted steps during the 1-min trial) using alternating arm swinging at different frequencies (*n* = 8 subjects, 24 trials total for each condition). **(B)** Arm EMG-based control of stepping in the exoskeleton by the healthy subject. Upper traces – rectified (gray) and low-pass filtered (black) EMGs of shoulder muscles. Each step duration and initiation were calculated and triggered based on the timing of the shoulder EMG peaks. Bottom traces – knee and hip joint angle kinematic patterns of eight consecutive steps along a 9-m walkway.

The suggested algorithm has also been implemented in the pilot experiments to trigger steps and control avatar walking (Figure 4A) and an exoskeleton during over-ground stepping (Figure 4B). Each trial consisted of the three locomotor-related phases controlled by the timing of EMG peaks: gait initiation, walking at a variable speed, and gait termination. In the absence of the EMG peak, the virtual avatar or exoskeleton did not produce further steps and gait termination was performed. The exoskeleton was tested only in one trained subject since typically the wearer has to use crutches to guarantee lateral stability (Wang et al., 2013). Nevertheless, this subject succeeded to use alternating EMG bursts of shoulder muscles to trigger 7–10 consecutive strides along a 8-m walkway (Figure 4B). Again, the percentage of successful trials for controlling virtual avatar at slow, self-selected, and fast frequency of arm movements was high (Figure 4A, lower panel) and similar to that found in the first experiment (Figure 2C) even though different subjects participated in the two protocols.

## Discussion

### Arm swinging during locomotion

Arm swing during human walking is not an entirely passive movement, as also shown by the observation that upper limb muscles show rhythmic activity contributing to arm swing (Ballesteros et al., 1965; Hogue, 1969; Ivanenko et al., 2006). Interestingly, rhythmic muscle activity continues to some extent even when the arm is immobilized (Kuhtz-Buschbeck and Jing, 2012). During walking, the EMG activity associated with arm swing is feeble compared with the EMG activity during maximum voluntary contractions. For instance, the mean amplitude values depend on speed and are lower at 4 km/h than at 6 km/h (Kuhtz-Buschbeck and Jing, 2012). However, even during fast walking (6 km/h), they remain well below 5% of maximum voluntary contraction, and yet, EMG patterns show prominent peaks around specific phases of the gait cycle. During running (7–12 km/h), the activity of shoulder muscles increases ~2–3 times relative to walking at 5 km/h (Cappellini et al., 2006) and also demonstrates a strong coupling between cervical and lumbosacral spinal motoneuron output (Ivanenko et al., 2008).

Previous studies describe this behavior both as a biomechanical advantage (Ortega et al., 2008; Park, 2008; Collins et al., 2009; Meyns et al., 2013) and a neural/evolutionary mechanism based on propriospinal pathways connecting distinct spinal segments, as seen in invertebrates and quadrupedal mammals (Falgairolle et al., 2006). Moreover, many features of quadrupedal arm–leg co-ordination are conserved across different locomotor tasks in human beings (Balter and Zehr, 2007; Patrick et al., 2012), including a reciprocal pattern of influences between the co-ordination of reaching and walking (Chiovetto and Giese, 2013), balance corrective responses (Forero and Misiaszek, 2014), or quadrupedal limb co-ordination during obstacle avoidance (Dietz and Michel, 2009).

### Gait assisted by EMG activity in upper limb muscles

Our study investigated the possibility of extracting spatiotemporal kinematic data from the gait-related rhythmic activity of arm muscles in order to provide a reliable control of walking. The results showed that, even with relatively low intensity of upper limb muscle activation, the temporal structure of EMG activity is sufficiently reliable to reproduce the spatiotemporal kinematic pattern of leg movements during walking at constant and variable speed (Figures 2 and 3). A comparison of actual and predicted stride leg kinematics showed a high degree of correspondence (*r* > 0.9). The suggested algorithm has been also implemented in pilot experiments for controlling avatar walking in a virtual reality environment and walking in the exoskeleton (Figure 4). Subjects were able to generate walking kinematics of the avatar for at least 1 min.

Myoelectric signals represent one of the measurable outputs of central nervous system activity. Thus, our study showed that this type of brain–computer interface can be used to link shoulder muscle EMG activity to leg movements (Figures 2 and 3) and to control ambulation within a virtual reality environment (Figure 4A), suggesting that a myoelectric-controlled lower extremity prosthesis for ambulation may be feasible. While the example in Figure 4A involves only four shoulder muscles and the example in Figure 4B involves two muscles, the implemented on-line algorithm can include the sum of bilateral EMGs of synergistic muscles (trapezius, latissimus dorsi, posterior and anterior portions of deltoid muscle, etc., individually adjusted for each patient to provide maximum comfort/efficiency) that are normally active during walking (Ivanenko et al., 2006; Kuhtz-Buschbeck and Jing, 2012). In fact, multi-muscle synergy-based control interface may be more efficient than a single-muscle control (Lunardini et al., 2014), as it also tended to be the case in our experiments when comparing predictions from two EMGs vs. four EMGs (Figure 2D), although further investigations are needed to compare different approaches, especially in neurological injuries with impaired inter-limb co-ordination.

Currently, many research projects are trying to apply novel, physiologically inspired control methods to provide an intuitive way for a patient to command an exoskeleton. Despite its deceiving simplicity, it is worth stressing that the implemented algorithm (Figure 1) takes into account a natural coupling of leg and arm movements during normal walking. Automaticity of arm swinging may be beneficial for the control of rhythmic leg movements, as opposed to a step-by-step voluntary control of muscles that are typically not involved in locomotion (e.g., when using push buttons or finger tapping for triggering stepping of a leg assistive robot that requires continuous cognitive resources, Lisi et al., 2014).

Finally, in addition to gait assistive aspects of exoskeleton robotic devices in severely paralyzed individuals, the proposed approach may also be beneficial for gait rehabilitation in less severe paresis of the lower limbs. Rhythmic upper limb muscle activation has an excitatory effect on lower limb muscle activation during locomotor-like tasks (Ferris et al., 2006; Massaad et al., 2014; Sylos-Labini et al., 2014a). To ensure a high level of EMG activity in deltoid (especially at a lower walking speed), the subjects performed slightly larger arm swinging than they usually do, which may require some additional voluntary control. Nevertheless, a special neural coupling occurs between arms and legs when arms move in alternation (Massaad et al., 2014), as well as active engagement of supraspinal motor areas reinforces CPG circuitry functioning (van den Brand et al., 2012; Solopova et al., 2014). This suggests that locomotor rehabilitation therapy after neurological injury should incorporate simultaneous arm and leg rhythmic exercise to take advantage of neural coupling. Such investigations stimulate new developments of neuroprosthetic technology and provide further insights into how we can integrate biological principles of control with an electromechanical exoskeleton that augments human performance for both gait assistive and rehabilitation technology.

## Conflict of Interest Statement

The authors declare that the research was conducted in the absence of any commercial or financial relationships that could be construed as a potential conflict of interest.
